# Utility of Cardiac Rehabilitation for Long-Term Outcomes in Patients with Hospital-Acquired Functional Decline after Cardiac Surgery: A Retrospective Study

**DOI:** 10.3390/jcm12124123

**Published:** 2023-06-18

**Authors:** Kotaro Hirakawa, Atsuko Nakayama, Kentaro Hori, Reina Uewaki, Tomoki Shimokawa, Mitsuaki Isobe

**Affiliations:** 1 Department of Rehabilitation, Sakakibara Heart Institute, Tokyo 183-0003, Japan; khori@shi.heart.or.jp (K.H.); ruewa@shi.heart.or.jp (R.U.); 2 Department of Cardiology, Sakakibara Heart Institute, Tokyo 183-0003, Japan; atsukonakanaka@gmail.com; 3 Department of Cardiovascular Surgery, Sakakibara Heart Institute, Tokyo 183-0003, Japan; tshimo@shi.heart.or.jp; 4 Sakakibara Heart Institute, Tokyo 183-0003, Japan; misobe@shi.heart.or.jp

**Keywords:** cardiac surgery, hospital-acquired functional decline, cardiac rehabilitation

## Abstract

Hospital-acquired functional decline is an important outcome that affects the long-term prognosis of patients after cardiac surgery. Phase II cardiac rehabilitation (CR) for outpatients is expected to improve prognosis; however, this is not clear in patients with hospital-acquired functional decline after cardiac surgery. Therefore, this study evaluated whether phase II CR improved the long-term prognosis of patients with hospital-acquired functional decline after cardiac surgery. This single-center, retrospective observational study included 2371 patients who required cardiac surgery. Hospital-acquired functional decline occurred in 377 patients (15.9%) after cardiac surgery. The mean follow-up period was 1219 ± 682 days in all patients, and there were 221 (9.3%) cases with major adverse cardiovascular events (MACE) after discharge during the follow-up period. The Kaplan–Meier survival curves indicated that hospital-acquired functional decline and non-phase II CR was associated with a higher incidence of MACE than other groups (log-rank, *p* < 0.001), additionally exhibiting prognosticating MACE in multivariate Cox regression analysis (HR, 1.59; 95% CI, 1.01–2.50; *p* = 0.047). Hospital-acquired functional decline after cardiac surgery and non-phase II CR were risk factors for MACE. The participation in phase II CR in patients with hospital-acquired functional decline after cardiac surgery could reduce the risk of MACE.

## 1. Introduction

Hospital-acquired functional decline is a decline in physical function and activities of daily living (ADL), which reportedly impacts the quality of life (QOL) and long-term prognosis of patients post their discharge [1,2]. It is associated with preoperative factors such as age, cognitive function, prehospital lifestyle, environment, and inpatient treatment factors such as bed-rest, malnutrition, and polypharmacy [3,4]. Hospital-acquired functional decline can affect the patients discharge from hospital and may require the introduction of new medical resources. Therefore, hospital-acquired functional decline is an important outcome measured at discharge in patients who have undergone cardiac surgery.

Phase II cardiac rehabilitation (CR) for outpatients discharged from acute care hospitals improves exercise capacity, QOL, cardiovascular-related death, and hospitalization in patients with cardiovascular diseases [5,6,7,8,9]. Therefore, outpatient CR is recommended by the guidelines of the Japanese Circulation Society [10]. However, all observational studies to date have involved patients with ischemic heart disease after coronary artery bypass graft surgery; no studies have been conducted on patients after cardiac surgery. Furthermore, postoperative cardiac surgery patients with hospital-acquired functional decline have been reported to have a higher risk of all-cause death and cardiovascular readmission after discharge [11], but the prognostic relevance of the introduction of phase II CR is unclear. In this study, we evaluated the role of phase II CR in determining the long-term prognosis of patients with hospital-acquired functional decline after cardiac surgery.

## 2. Materials and Methods

### 2.1. Participants

This study was conducted at a single cardiovascular center with the largest number of cardiovascular surgeries in Japan, retrospectively (1225 cardiovascular surgeries in 2021) [12]. This study included 2371 patients (median age 68 years; 36% female) who required cardiac surgery at our hospital between April 2014 and March 2020. The exclusion criteria were preoperative inability to walk independently, postoperative cerebral infarction, transfer to another hospital, in-hospital death, and lost to follow-up for more than 6 months.

### 2.2. Clinical Outcome

The primary outcome was major adverse cardiovascular events (MACE) occurring after discharge. MACE was characterized by the occurrence of any of the following in outpatient records: (1) stroke (including brain hemorrhage or infarction); (2) coronary revascularization by percutaneous coronary intervention; (3) either ST or non-ST segment elevation myocardial infarction; (4) heart failure-related hospitalization; and (5) cardiovascular death [13].

### 2.3. Definition of Hospital-Acquired Functional Decline

The Short Physical Performance Battery (SPPB) was used to assess physical function preoperatively and at discharge. The SPPB is a highly standardized geriatric physical functional test consisting of three items: (1) balance; (2) gait speed; and (3) chair stand. The balance tests included side-by-side, semi-tandem, and tandem standing, and patients were timed until they lost balance or 10s had elapsed. The gait speed test assessed the time required to walk 4 m and was performed at the patient’s usual pace. The chair-stand test, a pretest, was also performed, wherein patients were asked to fold their arms across their chest and stand up from a chair. If the pre-test was successful, the patients were asked to rise from the chair five times consecutively as quickly as possible. Each of the three subtests of the SPPB was scored from 0 to 4 and summed to obtain an SPPB score of 0–12 points [14]. Hospital-acquired functional decline was defined as a decrease in the SPPB score by at least 1 point at discharge from the preoperative score [15,16].

### 2.4. Cardiac Rehabilitation

Phase I CR was performed in accordance with the Guidelines for Rehabilitation in Cardiovascular Diseases established in 2021, complying with the inception and discontinuation criteria [10] including consultation with attending physicians. The days on which a patient was able to sit, stand, and practice walking were referred to as the onset of each activity.

Phase II CR was defined as participation in at least one month of the CR sessions after discharge. Phase II CR was performed once to three times a week under supervised and four to six times per week under unsupervised conditions for up to 5 months. Exercise consisted of preparatory and organized exercises such as stretching, aerobic exercise based on the exercise prescription obtained from cardiopulmonary exercise testing before starting, and resistance training on weight machines, as needed. Patients with low physical function and frailty were provided with individualized programs tailored to their goals. A multidisciplinary team implemented a comprehensive program for disease management, nutritional guidance, and psychological support.

### 2.5. Additional Assessments

The following data were collected from medical records: age, sex, body mass index (BMI), medical history of comorbidities, intraoperative records, Acute Physiology and Chronic Health Evaluation (APACHE) II score, operation and cardiopulmonary bypass (CPB) time, bleeding, duration of ventilator intubation, intra-aortic balloon pumping (IABP), continuous renal replacement therapy (CRRT), non-invasive positive pressure ventilation (NPPV), postoperative complications, length of intensive care unit (ICU) stay, length of hospital stay, postoperative blood biochemistry, and echocardiography examination results. Postoperative delirium was assessed in the ICU using the confusion assessment method [17].

### 2.6. Statistical Analysis

Continuous variables were expressed as a median (interquartile range [IQR]), and categorical variables were expressed as percentages. The patients were categorized according to the onset of hospital-acquired functional decline and whether they could participate in phase II CR. In addition, the Shapiro–Wilk test was used to verify normal distribution. For group comparisons, the Mann–Whitney U test was performed for continuous variables and the chi-squared test was used for categorical variables. Kaplan–Meier analysis was used to assess MACE and a log-rank test was performed to compare the groups. Cox regression analysis was performed with the presence of MACE as the dependent variable, as reported in previous studies and, significantly different variables in the univariate analysis were included as adjusted variables, such as female sex, age, BMI, hypertension, diabetes mellitus, chronic kidney disease, pre-SPPB, New York Heart Association (NYHA) ≥ III, chronic obstructive pulmonary disease, atrial fibrillation, operation time, bleeding, APACHE II score, invasive mechanical ventilation, NPPV, CRRT, delirium, length of hospital stay, left ventricular ejection fraction, hemoglobin, and, angiotensin-converting enzyme, estimated the hazard ratio (HR) and 95% CI for each combination of prognostic hospital-acquired functional decline and phase II CR. The level of significance was set at *p* ˂ 0.05, and all statistical analyses were performed using IBM SPSS Statistics version 22 (IBM Corp., Armonk, NY, USA).

## 3. Results

**Figure 1:**
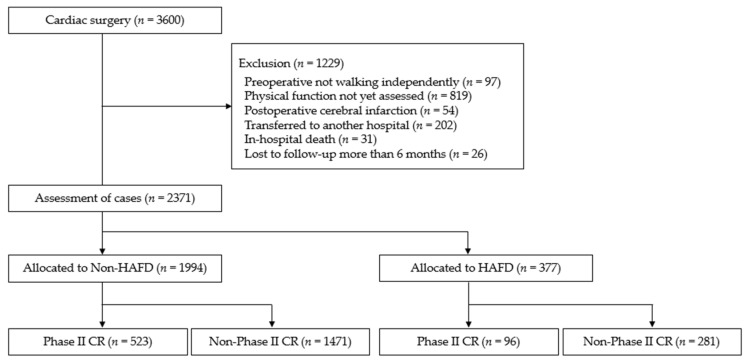
Schematic presentation of the study procedure. This study included 2371 patients who required cardiac surgery. HAFD, hospital-acquired functional decline, CR, cardiac rehabilitation.

Of the 2371 patients included in the analysis, 377 (15.9%) had hospital-acquired functional decline and 619 (26.1%) underwent phase II CR (Figure 1). The preoperative clinical characteristics, intraoperative findings, and postoperative courses of the patients are shown in Table 1 and Table 2. The hospital-acquired functional decline group was characterized by older age, multiple comorbidities, longer operative and ventilator intubation times, delayed postoperative rehabilitation progression, and a higher incidence of postoperative delirium than the non-hospital-acquired functional decline group. There were no significant differences in the clinical characteristics between the phase II and non-phase II CR groups in the hospital-acquired functional decline group.

**Figure 2:**
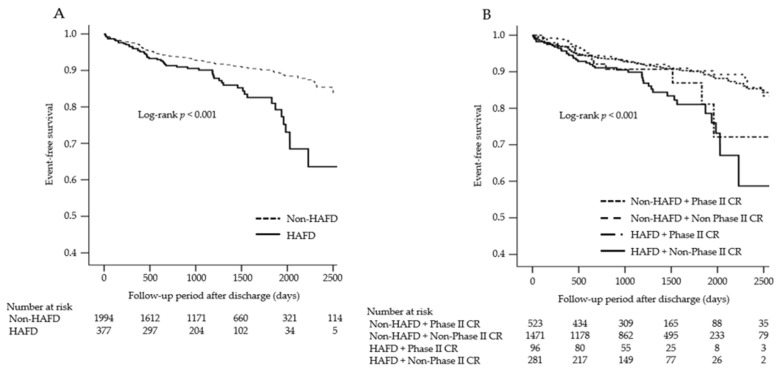
The Kaplan–Meier survival curves for MACE stratified according to hospital-acquired functional decline (**A**) and phase II CR (**B**). MACE, major adverse cardiovascular events; HAFD, hospital-acquired functional decline; CR, cardiac rehabilitation.

The mean follow-up duration was 1219 ± 682 days in all patients, and there were 221 (9.3%) cases of MACE occurring during the follow-up period after discharge. The incidence of MACE was 7.8% in the non-hospital-acquired functional decline and phase II CR, 8.6% in the non-hospital-acquired functional decline and non-phase II CR, 11.5% in the hospital-acquired functional decline and phase II CR, and 14.9% in the hospital-acquired functional decline and non-phase II CR groups. The Kaplan–Meier survival curves for MACE stratified according to hospital-acquired functional decline and phase II CR are shown in Figure 2. The results indicated that MACE were significantly associated with hospital-acquired functional decline (log-rank test, *p* < 0.001), and the hospital-acquired functional decline and non-phase II CR groups exhibited a higher incidence of MACE than the other groups (log-rank test, *p* < 0.001).

The results of the univariate and multivariate Cox regression analyses for MACE are shown in Table 3 and Table 4. Univariate Cox regression analysis revealed that age, sex, BMI, hypertension, chronic kidney disease, chronic heart failure, atrial fibrillation, chronic obstructive pulmonary disease, NYHA > III, operation time, CPB time, bleeding, duration of ventilator intubation, APACHE II score, NPPV, CRRT, delirium, length of hospital stay, postoperative rehabilitation progress, left ventricular ejection fraction, hemoglobin level, and pre- and post-SPPB were independent variables for MACE. Hospital-acquired functional decline and non-phase II was significantly predictive of MACE in multivariate analysis adjusting for these factors compared to hospital-acquired functional decline and non-phase II CR (HR, 1.59; 95% CI, 1.01–2.50; *p* = 0.047).

## 4. Discussion

We evaluated the relationship between hospital-acquired functional decline and phase II CR to prognosticate patients after cardiac surgery. Hospital-acquired functional decline and non-phase II CR were risk factors for MACE, which occurred in 9.3% of the patients after cardiac surgery.

### 4.1. Incidence of Hospital-Acquired Functional Decline and Phase II CR Participation Rates

Hospital-acquired functional decline has been reported in many previous studies [18] and is known to develop in 16–22% of patients undergoing cardiac surgery [11,19,20]. In this study, the incidence of hospital-acquired functional decline was 15.9%, which is similar to those of previous studies. In contrast, the participation rate in phase II CR was 26.1%. Notably, participation in outpatient CR with cardiovascular patients is low at 7–9% in Japan [21,22], and the institutional capacity, such as low number of cardiologists and hospitalized patients, has been cited as a factor in the declining number of participants [23]. This study was conducted at the cardiovascular center, which is one of the largest CR centers in Japan [12], and phases I and II provide seamless CR. Phase II CR is based on established evidence, and in recent years, phase I CR has been advocated, including early postoperative mobilization and neuromuscular electrical stimulation, which is an important treatment strategy for improving short- and long-term prognoses [24,25,26,27]. Therefore, phase I and II CR are necessary to prevent hospital-acquired functional decline and MACE. On the other hand, the non-phase II CR group included patients who did not agree to participate, and bias in adherence to medical recommendations between the phase II CR and non-phase II CR groups may have contributed to the results [28,29].

### 4.2. Relevance to MACE

In this study, the incidence of MACE was 9.3%, with a higher incidence reported in patients with hospital-acquired functional decline. Physical function decline at discharge is likely to persist long-term [30], leading to decreased physical activity [31]. Physical activity and sedentary behavior have been shown to be associated with negative health outcomes and are particularly pronounced in older adults [32]. The World Health Organization recommends increasing physical activity for good health, as a certain amount of physical activity can prevent cardiovascular diseases and improve mental function [33]. Hospital-acquired functional decline has been reported to be associated with decreased instrumental ADL and mental functioning after discharge, in addition to physical functional decline [34], which makes independent living more difficult. Thus, we believe that hospital-acquired functional decline results in a variety of situational changes after discharge, leading to poor prognosis.

In contrast, participation in phase II CR after the onset of hospital-acquired functional decline was associated with a reduced risk of MACE. Evidently, phase II CR has established its effectiveness in improving exercise tolerance and QOL along with preventing cardiovascular death and readmission in patients with coronary artery disease and heart failure [35,36]. Consequently, participation in phase II CR is recommended by the CR guidelines [10]. We could show that the positive effects of phase II CR were also seen in older patients, indicating the importance of exercise therapy that combines aerobic exercise and resistance training. Moreover, phase II CR contributes to the disease management program and is especially necessary for older patients who are unable to self-manage and make lifestyle modifications. Comprehensive CR is provided at this facility by a multidisciplinary team of nurses, registered dietitians, psychologists, and other professionals. The hospital-acquired functional decline group consisted of adults with an average age of 75 years, suggesting that the elderly may have improved prognosis if they participate in cardiac rehabilitation. On the other hand, the risk of developing MACE was higher in patients who were unable to participate in phase II CR after the onset of hospital-acquired functional decline. Older patients have characteristics that preclude their participation in phase II CR owing to multiple comorbidities, and cognitive and social aspects. Therefore, seamless follow-up after discharge through community collaboration is important to provide rehabilitation at home to patients who are unable to attend. In recent years, telerehabilitation using information and communication technology has been attracting attention. Home-based rehabilitation has been reported to be as effective as center-based cardiac rehabilitation programs [37], and it is expected that the development of MACE can be prevented through comprehensive programs for patients who are unable to attend.

### 4.3. Study Limitations

In this study, patients with cerebral infarction, which significantly impacts physical function, were excluded. Patients with these complications are more likely to be excluded from phase II CR because of their indications for a convalescent and rehabilitation hospital. In addition, patients who could not walk independently and were transferred to another hospital were unable to participate in phase II CR after discharge. Therefore, the results were based on patients who were eligible to participate in phase II CR. Additionally, there were patients whose physical function could not be assessed pre-operatively or at discharge because of emergency surgery, cognitive dysfunction, or absence of an assessor.

First, we excluded patients who did not walk independently and those who had not been subjected to phase II CR. Therefore, patients with preoperative frailty and sarcopenia with significant declines in physical or cognitive function were not included, as the MACE-related factors in such patients are not clear. Second, the living environment, including exercise habits, of patients who did not participate in phase II CR is unknown. These patients may exercise after discharge from gyms or nursing care services. In addition, changes in physical function and ADL after discharge could not be tracked.

In future, the authors should discuss these results and how they can be interpreted from the perspectives of previous studies and working hypotheses. These findings and their implications should be discussed in the broadest possible context. Future research directions have also been highlighted.

## 5. Conclusions

We found that hospital-acquired functional decline after cardiac surgery and not participating in phase II CR were associated with a higher risk of developing MACE. The participation in phase II CR of patients with hospital-acquired functional decline after cardiac surgery is expected to reduce the risk of MACE.

## Figures and Tables

**Table 1:** Clinical characteristics in HAFD (HAFD, hospital-acquired functional decline; BMI, body mass index; NYHA, New York Heart Association; CABG, coronary artery bypass grafting; CPB, cardiopulmonary bypass; IMV, invasive mechanical ventilation; APACHE, Acute Physiology and Chronic Health Evaluation; IABP, intra-aortic balloon pumping; NPPV, non-invasive positive pressure ventilation; CRRT, continuous renal replacement therapy; LVEF, left ventricular ejection fraction; CRP, C-reactive protein; ACE, angiotensin-converting enzyme; ARB, angiotensin II receptor blocker; CCB, calcium channel blocker; SPPB, Short Physical Performance Battery).

	Non-HAFD	HAFD	*p*-Value
	(*n* = 1994)	(*n* = 377)	
Age, years	66 (55–74)	75 (69–79)	<0.001
≥65 years, *n* (%)	1115 (55.9)	318 (84.4)	<0.001
Female, *n* (%)	655 (32.8)	187 (49.6)	<0.001
BMI, kg/m^2^	22.8 (20.7–25.2)	22.8 (20.6–25.2)	0.27
Comorbidity, *n* (%)			
Hypertension	1025 (51.4)	223 (59.2)	<0.01
Diabetes mellitus	361 (18.1)	114 (30.2)	<0.001
Chronic kidney disease	170 (8.5)	56 (14.9)	<0.001
Chronic heart failure	223 (11.2)	60 (15.9)	<0.01
Chronic obstructive pulmonary disease	36 (1.8)	6 (1.6)	0.77
Atrial fibrillation	294 (14.7)	71 (18.8)	0.04
Anemia	299 (15.0)	60 (15.9)	0.65
NYHA class ≥ III, *n* (%)	126 (6.3)	41 (10.9)	<0.01
Surgery type, *n* (%)			<0.01
CABG	466 (23.4)	108 (28.6)	
Valve	571 (28.6)	85 (22.5)	
Multiple	825 (41.4)	170 (45.1)	
Other	132 (6.6)	14 (3.7)	
Operation time, min	259 (210–318)	283 (225–354)	<0.001
CPB time, min	146 (114–185)	145 (114–187)	0.09
Bleeding, ml	140 (80–240)	160 (90–280)	<0.01
IMV, h	12 (10–18)	15 (11–21)	<0.001
APACHE II score, points	11 (9–13)	13 (11–14)	<0.001
IABP, *n* (%)	8 (0.4)	1 (0.3)	0.57
NPPV, *n* (%)	51 (2.6)	25 (6.6)	<0.001
CRRT, *n* (%)	8 (0.4)	4 (1.1)	0.11
Pneumonia, *n* (%)	8 (0.4)	4 (1.1)	0.11
Delirium, *n* (%)	81 (4.1)	53 (14.1)	<0.001
Postoperative rehabilitation, days			
Standing	1 (1–1)	1 (1–2)	<0.001
Walking	2 (1–2)	2 (1–3)	<0.001
LVEF, %	57.0 (51.0–61.2)	58.5 (51.3–62.5)	0.01
Hemoglobin, g/dL	11.2 (10.1–12.3)	10.7 (9.7–11.6)	<0.001
Albumin, g/dL	3.3 (3.1–3.6)	3.3 (2.9–3.4)	0.08
Creatinine, mg/dL	0.82 (0.69–0.95)	0.80 (0.65–0.98)	0.31
CRP, mg/dL	2.11 (1.18–3.71)	2.23 (1.27–3.74)	0.42
β-blocker, *n* (%)	1291 (64.7)	214 (56.8)	<0.01
ACE inhibitor, *n* (%)	144 (7.2)	25 (6.6)	0.68
ARB, *n* (%)	482 (24.2)	92 (24.4)	0.92
CCB, *n* (%)	665 (33.4)	124 (32.9)	0.57
SPPB, points			
Preoperative	12 (12–12)	12 (11–12)	<0.001
Discharge	12 (12–12)	10 (9–11)	<0.001
Hospital stay, days	10 (8–13)	11 (9–15)	<0.001

**Table 2:** Clinical characteristics in HAFD and phase II CR (HAFD, hospital-acquired functional decline; CR, cardiac rehabilitation; BMI, body mass index; NYHA, New York Heart Association; CABG, coronary artery bypass grafting; CPB, cardiopulmonary bypass; IMV, invasive mechanical ventilation; APACHE, Acute Physiology and Chronic Health Evaluation; IABP, intra-aortic balloon pumping; NPPV, non-invasive positive pressure ventilation; CRRT, continuous renal replacement therapy; LVEF, left ventricular ejection fraction; CRP, C-reactive protein; ACE, Angiotensin-converting enzyme; ARB, angiotensin II receptor blocker; CCB, calcium channel blocker; SPPB, Short Physical Performance Battery).

	Non-HAFD		*p*-Value	HAFD		*p*-Value
	Phase II CR	Non-Phase II CR		Phase II CR	Non-Phase II CR	
	(*n* = 523)	(*n* = 1471)		(*n* = 96)	(*n* = 281)	
Age, years	68 (60–74)	66 (53–74)	<0.01	76 (70–79)	75 (68–80)	0.97
≥65 years, *n* (%)	334 (63.9)	781 (53.1)	<0.001	83 (86.5)	235 (83.6)	0.51
Female, *n* (%)	164 (31.4)	491 (33.4)	0.40	42 (43.8)	145 (51.6)	0.18
BMI, kg/m^2^	23.0 (21.0–25.2)	22.8 (20.6–25.2)	0.15	23.3 (21.3–25.1)	22.4 (20.3–24.8)	0.06
Comorbidity, *n* (%)						
Hypertension	291 (55.6)	734 (49.9)	0.02	64 (66.7)	159 (56.6)	0.08
Diabetes mellitus	127 (24.3)	234 (15.9)	<0.001	30 (31.3)	84 (29.9)	0.80
Chronic kidney disease	38 (7.3)	132 (9.0)	0.23	12 (12.5)	44 (15.7)	0.45
Chronic heart failure	52 (9.9)	171 (11.6)	0.29	10 (10.4)	50 (17.8)	0.09
Chronic obstructive pulmonary disease	9 (1.7)	27 (1.8)	0.87	0 (0.0)	6 (2.1)	0.17
Atrial fibrillation	65 (12.4)	229 (15.6)	0.08	16 (16.7)	55 (19.6)	0.53
Anemia	63 (12.0)	236 (16.1)	0.03	10 (10.4)	50 (17.8)	0.09
NYHA class ≥ III, *n* (%)	23 (4.4)	103 (7.0)	0.04	8 (8.3)	33 (11.7)	0.35
Surgery type, *n* (%)			<0.001			<0.001
CABG	178 (34.0)	288 (19.6)		44 (45.8)	64 (22.8)	
Valve	140 (26.8)	431 (29.3)		12 (12.5)	73 (26.0)	
Multiple	191 (36.5)	634 (43.1)		38 (39.6)	132 (47.0)	
Other	14 (2.7)	118 (8.0)		2 (2.1)	12 (4.3)	
Operation time, min	265 (215–320)	255 (208–315)	0.09	295 (236–359)	280 (219–353)	0.34
CPB time, min	147 (113–281)	145 (114–187)	0.64	150 (111–201)	152 (116–207)	0.46
Bleeding, ml	150 (80–250)	135 (80–240)	0.11	160 (90–289)	160 (90–280)	0.86
IMV, h	12 (10–18)	12 (10–19)	0.62	15 (11–21)	15 (11–21)	0.67
APACHE II score, points	12 (10–13)	11 (9–13)	0.02	13 (11–14)	13 (11–14)	0.94
IABP, *n* (%)	4 (0.8)	4 (0.3)	0.13	0 (0.0)	1 (0.4)	0.75
NPPV, *n* (%)	11 (2.1)	40 (2.7)	0.44	6 (6.3)	19 (6.8)	0.86
CRRT, *n* (%)	2 (0.4)	6 (0.4)	0.65	0 (0.0)	4 (1.4)	0.31
Pneumonia, *n* (%)	2 (0.4)	6 (0.4)	0.65	0 (0.0)	4 (1.4)	0.31
Delirium, *n* (%)	17 (3.3)	64 (4.4)	0.27	10 (10.4)	43 (15.3)	0.23
Postoperative rehabilitation, days						
Standing	1 (1–1)	1 (1–1)	0.49	1 (1–1)	1 (1–1)	0.19
Walking	2 (1–2)	2 (1–2)	0.97	2 (1–3)	1 (1–3)	0.27
LVEF, %	57.7 (51.8–61.8)	56.7 (50.8–61.0)	0.01	58.5 (53.8–62.9)	58.6 (50.7–62.4)	0.27
Hemoglobin, g/dL	11.2 (10.2–12.3)	11.2 (10.1–12.3)	0.47	10.6 (9.6–11.6)	10.7 (9.8–11.6)	0.61
Albumin, g/dL	3.3 (3.1–3.6)	3.4 (3.1–3.7)	0.38	3.3 (3.1–3.6)	3.3 (2.9–3.4)	0.85
Creatinine, mg/dL	0.82 (0.70–0.96)	0.82 (0.69–0.95)	0.74	0.80 (0.63–0.98)	0.81 (0.65–0.99)	0.75
CRP, mg/dL	2.01 (1.16–3.33)	2.15 (1.19–3.80)	0.14	2.45 (1.24–3.86)	2.15 (1.27–3.65)	0.25
β–blocker, *n* (%)	241 (46.1)	1050 (71.4)	<0.001	40 (41.7)	174 (61.9)	<0.01
ACE inhibitor, *n* (%)	20 (3.8)	124 (8.4)	<0.001	4 (4.2)	21 (7.5)	0.26
ARB, *n* (%)	62 (11.9)	420 (28.6)	<0.001	10 (10.4)	82 (29.2)	<0.001
CCB, *n* (%)	135 (25.8)	534 (36.3)	<0.001	13 (13.5)	107 (38.1)	<0.001
SPPB, points						
Preoperative	12 (12–12)	12 (12–12)	0.36	12 (12–12)	12 (11–12)	0.06
Discharge	12 (12–12)	12 (12–12)	0.18	10 (9–11)	10 (9–11)	0.68
Hospital stay, days	10 (9–13)	10 (8–13)	0.94	10 (9–13)	11 (9–16)	0.01

**Table 3:** Univariate analyses for major adverse cardiovascular events (BMI, body mass index; NYHA, New York Heart Association; CABG, coronary artery bypass grafting; CPB, cardiopulmonary bypass; IMV, invasive mechanical ventilation; APACHE, Acute Physiology and Chronic Health Evaluation; IABP, intra-aortic balloon pumping; NPPV, non-invasive positive pressure ventilation; CRRT, continuous renal replacement therapy; LVEF, left ventricular ejection fraction; CRP, C-reactive protein; ACE, angiotensin-converting enzyme; ARB, angiotensin II receptor blocker; CCB, calcium channel blocker; SPPB, Short Physical Performance Battery; HAFD, hospital-acquired functional decline; CR, cardiac rehabilitation).

	Overall	Univariate		
	(*n* = 2371)	HR	95% CI	*p*-Value
Age, years	68 (57–75)	1.036	1.024–1.048	<0.001
≥65 years, *n* (%)	1433 (60.4)	1.875	1.395–2.519	<0.001
Female, *n* (%)	842 (35.5)	0.963	0.729–1.272	0.79
BMI, kg/m^2^	22.7 (20.7–25.1)	1.030	0.991–1.070	0.13
Comorbidity, *n* (%)				
Hypertension	1248 (52.6)	1.639	1.247–2.155	<0.001
Diabetes mellitus	475 (20.0)	1.450	1.066–1.971	0.02
Chronic kidney disease	226 (9.5)	1.892	1.318–2.715	<0.01
Chronic heart failure	462 (19.5)	1.732	1.241–2.417	<0.01
Atrial fibrillation	365 (15.4)	1.730	1.264–2.366	<0.01
Anemia	359 (15.1)	1.277	0.907–1.796	0.16
Chronic obstructive pulmonary disease	42 (1.8)	2.394	1.269–4.516	<0.01
NYHA class ≥ III, *n* (%)	167 (7.0)	1.768	1.157–2.703	<0.01
Surgery type, *n* (%)				
CABG	574 (24.2)			
Valve	656 (27.7)			
Multiple	995 (42.0)			
Other	146 (6.2)			
Operation time, min	262 (212–324)	1.004	1.003–1.005	<0.001
CPB time, min	146 (114–188)	1.004	1.002–1.006	<0.001
Bleeding, mL	140 (80–250)	1.001	1.001–1.001	<0.001
IMV, h	13 (10–19)	1.018	1.011–1.024	<0.001
APACHE II score, points	12 (10–13)	1.120	1.073–1.169	<0.001
IABP, *n* (%)	9 (0.4)	2.123	0.527–8.561	0.29
NPPV, *n* (%)	76 (3.2)	2.470	1.484–4.110	<0.01
CRRT, *n* (%)	15 (0.6)	7.475	3.318–16.838	<0.001
Pneumonia, *n* (%)	12 (0.5)	2.299	0.571–9.258	0.24
Delirium, *n* (%)	134 (5.7)	2.254	1.434–3.542	<0.001
Hospital stay, days	10 (9–13)	1.037	1.025–1.049	<0.001
Postoperative rehabilitation, days				
Standing	1 (1–1)	1.094	1.033–1.158	<0.01
Walking	2 (1–2)	1.114	1.071–1.159	<0.001
LVEF, %	57.2 (51.0–61.4)	0.980	0.967–0.992	<0.01
Hemoglobin, g/dL	11.1 (10.1–12.2)	0.814	0.743–0.893	<0.001
Albumin, g/dL	3.3 (3.1–3.6)	0.535	0.155–1.848	0.32
Creatinine, mg/dL	0.81 (0.69–0.95)	1.103	0.980–1.241	0.11
CRP, mg/dL	2.13 (1.20–3.71)	0.955	0.897–1.015	0.14
β–blocker, *n* (%)	1505 (63.5)	0.921	0.673–1.261	0.61
ACE inhibitor, *n* (%)	169 (7.1)	0.579	0.382–0.877	0.01
ARB, *n* (%)	574 (24.2)	0.828	0.603–1.137	0.24
CCB, *n* (%)	789 (33.3)	0.829	0.618–1.112	0.21
Pre SPPB, points	12 (12–12)	0.810	0.748–0.877	<0.001
Post SPPB, points	12 (11–12)	0.830	0.777–0.886	<0.001
HAFD, *n* (%)	337 (15.9)	1.886	1.383–2.572	<0.001
Phase II CR, *n* (%)	619 (26.1)	0.850	0.623–1.160	0.31

**Table 4:** Multivariate analyses for major adverse cardiovascular events (HAFD, hospital-acquired functional decline; CR, cardiac rehabilitation; BMI, body mass index; HT, hypertension; DM, diabetes mellitus; CKD, chronic kidney disease; SPPB, Short Physical Performance Battery; NYHA, New York Heart Association; COPD, chronic obstructive pulmonary disease; APACHE, Acute Physiology and Chronic Health Evaluation; IMV, invasive mechanical ventilation; NPPV, non-invasive positive pressure ventilation; CRRT, continuous renal replacement therapy; LVEF, left ventricular ejection fraction; ACE, angiotensin-converting enzyme. * Model1 was adjusted for female sex, age, BMI, HT, DM, and CKD. † Model2 adjusted: Model1 + pre-SPPB, NYHA ≥ III, COPD, atrial fibrillation, operation time, bleeding, APACHE II score, IMV, NPPV, CRRT, delirium, length of hospital stay, LVEF, hemoglobin, ACE).

	Univariate			Multivariate *			Multivariate †		
	HR	95% CI	*p*-Value	HR	95% CI	*p*-Value	HR	95% CI	*p*-Value
Non-HAFD + Phase II CR	1.000			1.000			1.000		
Non-HAFD + Non-Phase II CR	1.118	0.786–1.591	0.53	1.187	0.833–1.691	0.34	1.132	0.783–1.635	0.51
HAFD + Phase II CR	1.580	0.812–3.074	0.18	1.354	0.692–2.647	0.38	1.265	0.639–2.505	0.50
HAFD + Non-Phase II CR	2.225	1.444–3.428	<0.001	1.981	1.275–3.078	<0.01	1.607	1.018–2.538	<0.05

## Data Availability

The datasets used and analyzed in the current study are available from the corresponding author upon reasonable request.
